# South-East Asia regional neglected tropical disease framework: improving control of mycetoma, chromoblastomycosis, and sporotrichosis

**DOI:** 10.1016/j.lansea.2025.100561

**Published:** 2025-03-27

**Authors:** Dallas J. Smith, Hardyanto Soebono, Niraj Parajuli, Marlous L. Grijsen, Alyson M. Cavanaugh, Tom Chiller, Prajwal Pudasaini, Terlinda C. Barros, Arunaloke Chakrabarti

**Affiliations:** a Mycotic Diseases Branch, Centers for Disease Control and Prevention, Atlanta, GA, USA; b Department of Dermatology and Venereology, Faculty of Medicine, Public Health, and Nursing, Universitas Gadjah Mada, Yogyakarta, Indonesia; c Center for Tropical Medicine, Universitas Gadjah Mada, Yogyakarta, Indonesia; d Department of Dermatology & Venereology, National Academy of Medical Sciences, Bir Hospital, Kathmandu, Nepal; e Oxford University Clinical Research Unit Indonesia, Faculty of Medicine Universitas Indonesia, Jakarta, Indonesia; f Centre for Tropical Medicine and Global Health, Nuffield Department of Medicine, University of Oxford, United Kingdom; g Department of Dermatology, Civil Service Hospital, Government of Nepal, Kathmandu, Nepal; h Hospital Nacional Guido Valadares, Dili, Timor-Leste; i Doodhadhari Burfani Hospital and Research Institute, Haridwar, India

**Keywords:** Neglected tropical diseases, Fungi, Chromoblastomycosis, Mycetoma, Sporotrichosis, South-East Asia

## Abstract

Mycetoma, chromoblastomycosis, and sporotrichosis are fungal neglected tropical diseases (NTDs) recognized by the World Health Organization. These implantation diseases cause substantial morbidity, disability, decreased quality of life, and can lead to long-term complications including tissue fibrosis, skin cancer, and amputation. The 2024–2030 South-East Asia Regional NTD Strategic Framework includes mycetoma but neglects the full extent of mycetoma endemicity in the region. Furthermore, the framework excludes chromoblastomycosis and sporotrichosis. We describe the data demonstrating fungal NTDs being of public health concern in this region and more widely distributed than acknowledged in the framework. Additionally, we propose modifications to public health interventions and services for fungal NTDs including an active case search approach through community health workers. Severe disease from fungal NTDs in South-East Asia can be eliminated by improving burden data quality, early diagnosis, accessible treatment, and integration with other common and neglected skin diseases.

## Introduction to fungal neglected tropical diseases

Neglected tropical diseases (NTD) are a diverse group of World Health Organization (WHO)-recognized diseases caused by bacteria, fungi, parasites, toxins, or viruses that disproportionately impact impoverished communities in tropical areas.^1^ NTDs often cause disability and are associated with decreased quality of life and stigmatization. At least 13 of these diseases have skin-manifestations and are considered skin-NTDs.^1^^,^^2^ Skin-NTDs require specialized skills for accurate identification and management, regularly involving years of resource-intensive care. Gaps in awareness, capacity, training, and health care infrastructure in rural settings, where these diseases are more common, prevent effective clinical and public health interventions.^3^ Three diseases with fungal components (mycetoma, chromoblastomycosis, and sporotrichosis) are included in the 2021–2030 WHO Global NTD roadmap and are skin-NTDs.^1^^,^^3^

In recent years, national and global efforts have placed more focus on improving diagnostics, treatment, and surveillance of skin NTDs in the context of health care strengthening. Unfortunately, fungal NTDs continue to be underrecognized as robust disease burden is lacking, making these diseases some of the most neglected of the NTDs.^4^ The strategic framework for NTDs published by the WHO South-East Asia regional office demonstrates the limited data, awareness and focus on fungal NTDs.^5^ This report summarizes information on fungal NTDs, evidence regarding their public health importance, and interventions for consideration by countries and regional partners in the South-East Asia region.

## What are the fungal NTDs?

Mycetoma, chromoblastomycosis, and sporotrichosis are considered implantation diseases that are acquired after the pathogen enters the skin after trauma, and commonly affect low-income agrarian populations.^6^ Paracoccidioidomycosis, although often involving skin manifestations, primarily is acquired through the inhalation of fungal spores from the environment; this disease is only endemic to the Americas.^7^

Mycetoma can be caused by several different types of bacteria and fungi. It is characterized by progressively debilitating subcutaneous tumor-like lesions, usually on the extremities.^8^ Infections are thought to occur most commonly in the Mycetoma Belt between the latitudes of 15° S and 30° N, though some evidence suggests this range may be expanding. Fungal mycetoma (eumycetoma) and bacterial mycetoma (actinomycetoma) both cause substantial disease in Africa, Asia, and South America, while actinomycetoma accounts for the majority of cases in Mexico and Central America. Eumycetoma is most commonly caused by *Madurella mycetomatis* and *Falciformispora senegalensis* while actinomycetoma is commonly caused by *Nocardia brasiliensis* and *Actinomadura madurae*^8^^,^^9^ Proper treatment requires an accurate diagnosis that distinguishes eumycetoma from actinomycetoma. Eumycetoma is more difficult to treat and has lower cure rates.^8^ Mycetoma, without timely diagnosis and treatment, often leads to tissue destruction, amputation, and loss of mobility.^8^

Chromoblastomycosis is a sporadically occurring infection seen in tropical and subtropical climates caused by a number of different dematiaceous fungi, the most common of which are *Fonsecaea pedrosoi*, *F. monophora*, *F. nubica*, *Cladophialophora carrionii*, *Rhinocladiella aquaspersa*, and *Phialophora verrucosa*.^10^ Chromoblastomycosis can cause disability due to limb enlargement, which may lead to inability to work and considerable social stigma. Rarely, the fungi that cause chromoblastomycosis can infect other organs, such as the brain, or lead to skin cancer.^11^ Patients with large or extensive chromoblastomycosis and limb swelling often require over a year of treatment and there may be residual lymphoedema in such cases.^10^ Itraconazole or terbinafine are typically first-line treatments, although no formal treatment guidelines exist.

Sporotrichosis is considered the most common and widespread implantation mycosis, although, more common in tropical and subtropical regions. It is caused by a number of different fungi of the genus *Sporothrix*, including *Sporothrix schenckii*, *Sporothrix globosa*, and *Sporothrix brasiliensis*.^12^ The fungi exist naturally on plant materials and can invade deeper layers of the skin after trauma. *S. brasiliensis* can infect cats and other mammals, with the potential to spread to humans from animals.^13^ Sporotrichosis leads to painful and unsightly skin lesions predominately over exposed body sites leading to social stigma and socio-economic consequences. Rarely, deep forms of sporotrichosis can occur that can lead to arthritis, respiratory infection and even meningitis.^14^

## Regional strategic framework for sustaining, accelerating and innovating to end neglected tropical diseases in the South-East Asia region 2024–2030

The WHO South-East Asia regional office has published a framework to end NTDs in the region. This framework aims to guide, coordinate, and integrate efforts among Member States, WHO, and partners in the region to sustain progress, accelerate actions, and foster innovative approaches to effectively implement the WHO Global NTD Roadmap 2021–2030.^1^^,^^5^ The framework describes overarching, cross-cutting, and disease-specific indicators for fifteen NTDs deemed of significant public health concern to the region. However, of the four WHO fungal NTDs, only mycetoma was included in this regional priority list. Only three out of eleven countries were noted to have reported cases of mycetoma.^5^

In the framework, each NTD had strategic priorities outlined for Member States and WHO and partners to address. The strategic priorities for Member States for mycetoma were to 1) establish surveillance to ensure reporting of cases through the health system to determine the need for public health interventions and classify higher risk geographic regions and 2) roll out an integrated approach to skin NTDs through strengthening the capacity for detection, diagnosis and management of cases. For strategic mycetoma priorities for WHO and partners, 1) finalize the regional integrated skin NTD toolkit and implement across the region and 2) mobilize resources and support countries in capacity-building and implementation of the strategic priorities. It was noted that zero countries include mycetoma in national control programs and surveillance systems with a 2026 milestone of one country and a 2030 target of two countries.

Specific public health interventions and services for mycetoma at community and health-facility level included surveillance and monitoring and evaluation (passive surveillance) intensified disease management (case management/treatment, wound care, surgery, rehabilitation), and community interventions (social and behavioral change communication, community hygiene and environmental management). Microscopy, polymerase chain reaction, and imaging were included for required diagnostics; for therapeutics, only antifungals were included.

## Reported cases of mycetoma in South-East Asia region countries

The true burden of mycetoma across the region is not well understood due to limited country-level or regional surveillance. In the published framework, India, Thailand, and Timor-Leste were highlighted as having reported cases of mycetoma. A 2013 literature review found 1392 cases of mycetoma published from India, the country with the third highest number of cases globally.^15^ Thailand had 17 reported cases and was the only country that reported mycetoma cases in the 2017 global WHO survey on mycetoma from the region.^16^ Several cases have recently been reported from Timor-Leste.^17^

However, case reports of mycetoma have been published in other countries in South-East Asia as well. In Indonesia for example, a single-center case series and several locally acquired cases have been published.^18^, ^19^, ^20^, ^21^ Nepal has reported at least two cases of mycetoma; although it is believed to be more widespread across the country, particularly given the high prevalence in northern India.^22^, ^23^, ^24^, ^25^ In Sri Lanka, several cases have been reported and the Ministry of Health's Epidemiology Unit has included information on mycetoma in a weekly epidemiological report.^26^, ^27^, ^28^, ^29^ These cases were not reported to the WHO and were not included in the WHO South-East Asia NTD framework.

In many South-East Asian countries, NTDs are often integrated into broader programs, such as vector-borne diseases or zoonotic diseases, often under departments like the Communicable Diseases Directorate. Mycetoma may not neatly fit into these existing programs, and may not be prioritized as it often causes chronic disabilities and lower quality of life rather than being associated with mortality.^30^ With funding shortfalls and competing health priorities, MOH approaches are absent to drive an accurate estimate of mycetoma prevalence and endemicity remains unknown. To bridge this gap, a cross-cutting approach to skin NTD surveillance is essential. Leveraging existing programs (e.g., WASH, dermatology, surgery), while ensuring mycetoma and other skin NTD are included, can help generate more accurate data and provide a better understanding of the true burden of these conditions.

## Have chromoblastomycosis and sporotrichosis been neglected in the priority lists in South-East Asia?

Chromoblastomycosis and sporotrichosis were notably missing from the regional strategic framework on NTDs. Recent studies have demonstrated that chromoblastomycosis and sporotrichosis are endemic to South-East Asia. A literature review to estimate a global burden of chromoblastomycosis found 169 cases from India, 71 cases from Sri Lanka, 15 cases from Nepal, 14 cases from Thailand, 13 cases from Indonesia, and one case from Bangladesh.^31^ Teledermatology has identified additional cases of chromoblastomycosis in Indonesia in remote areas.^32^^,^^33^

Historically, human sporotrichosis cases has been sporadically reported in several parts of South-East Asia (e.g., India, Nepal, Bangladesh, Indonesia) suggesting that the disease is likely present across the entire region.^12^^,^^34^, ^35^, ^36^, ^37^ Concerningly, in recent years, the spread of zoonotic sporotrichosis by cats has increased substantially for unknown reasons, particularly in Thailand where a single center reported 32 cases associated with cats since 2018.^38^^,^^39^

The burden of chromoblastomycosis and sporotrichosis remains largely unknown, similar to mycetoma, due to the lack of surveillance systems. Data from published reports likely represent a substantial underestimation of the true burden of disease. Limited awareness of these diseases by both the healthcare workers and national programs represents a challenge to accurately diagnosing and monitoring the prevalence of these diseases in the region.

## A path forward for public health interventions and services for fungal neglected tropical diseases

Mycetoma, chromoblastomycosis, and sporotrichosis are each targeted for control in the WHO Global NTD Roadmap 2023–2030.^1^ The organisms that cause fungal NTDs live in the environment, and eradication or elimination of these diseases is not realistic. However, an integrated approach promoting early diagnosis and effective management may reduce severe disease (e.g., tissue fibrosis, dissemination, skin cancer, amputation) and morbidity substantially.^40^ This can be achieved by training healthcare professionals, early identification in community settings through active case detection, strengthening diagnostic capacity and infrastructure, integrating One Health principles, and ensuring sustained access to affordable treatment. We applaud the comprehensive, regional approach taken to address NTDs in South-East Asia but propose modifications to the public health interventions and services for fungal NTDs.

A noticeable gap in this framework for public health surveillance interventions for mycetoma, chromoblastomycosis, and sporotrichosis was active case detection and population surveys. Relying solely on passive surveillance may result in late-stage disease identification reducing successful treatment and increasing risk of developing complications requiring advanced surgery or amputation.^41^^,^^42^ Active case finding enables early identification, facilitating prompt access to treatment and wound management. A clinical trial for eumycetoma in Sudan using active case detection to identify individuals found a 75% cure rate for individuals with smaller lesions treated with itraconazole, which is considered standard of care for eumycetoma.^40^ Community health workers can be trained to conduct active case detection for mycetoma, chromoblastomycosis, sporotrichosis and other common and neglected skin diseases and refer people to the appropriate level of care.^43^^,^^44^ These programs do not need to exist in a vacuum but can be integrated with other skin-NTDs to synergize resources, expertise, personnel.^3^ Active case finding has been employed for other skin-NTDs, like leprosy and leishmaniasis, in the region and could be expanded to include fungal NTDs if adequate funding is available.^45^, ^46^, ^47^, ^48^ In parallel, strengthening passive case detection within the healthcare system could help identify geographic areas with higher disease prevalence, guiding tailored public health interventions, such as community outreach and clinical and laboratory education.

Zoonotic sporotrichosis requires a One Health approach that will require collaboration between human clinicians, veterinarians, and public health officials.^13^ A novel species, *S. brasiliensis*, has emerged in Brazil causing an epidemic of zoonotic sporotrichosis via cats that has spread to neighboring countries and even to the United Kingdom.^49^ In Rio de Janeiro, Brazil, at least 14,490 human cases and 20,202 cat cases of sporotrichosis were reported to the State Health Department from 2011 to April 2023.^50^ Whole genome sequencing has revealed that this species emerged independently, multiple times in Brazil, suggesting a possibility of occurrence in other places in the world.^51^ More data is needed to determine the exact cause of the zoonotic sporotrichosis outbreak in Thailand, and the extent of zoonotic spread in other parts of South-East Asia. Additionally, studies assessing the impact of veterinarian treatment of cats in preventing transmission to humans can be conducted to inform policy decisions. A framework can be adopted similar to that from Brazil where public health authorities and clinicians are focusing on treating cats to prevent further transmission with some Brazilian states providing free treatment. Treatment of cats could fall under vector control and veterinarian public health in the South-East Asia NTD framework alongside use of personal protective equipment for veterinarians and cremation of cats who died with sporotrichosis.^13^ One Health interventions for mycetoma, chromoblastomycosis, and sapronotic sporotrichosis include utilizing non-culture based environmental sampling techniques to better understand geographical distribution and niches and conducting studies on prevention of fungal implantation.^10^^,^^52^

The South-East Asia NTD framework emphasizes strengthening laboratory and diagnostic capacity at the country level. An assessment on the availability of fungal testing in the entire Asia region was conducted and found large variability in the ability to test fungal infections.^53^ Countries in South-East Asia that participated in the survey (i.e., Bangladesh, India, Indonesia, Thailand) had less capacity to conduct direct microscopy, culture, molecular tests, and antifungal susceptibility testing than the average in Asia, excluding India which had higher capacity. Additionally, most respondents were from university hospitals with high annual budget, likely not reflecting the true diagnostic capacity of the countries, especially in rural areas where fungal NTDs are more common. We propose that the South-East Asia NTD framework specifically encourage improved fungal disease diagnostics and training at a country-level focusing on direct microscopy for all fungal NTDs in rural and primary health care settings. Additionally, portable ultrasound technology has shown promise in decentralized settings to, not only diagnose mycetoma, but distinguish between actinomycetoma and eumycetoma and could be integrated into primary health settings in South-East Asia.^54^

Population surveys for fungal NTDs can help forecast medication needs in communities bearing a high prevalence of disease. Itraconazole is considered standard of care for eumycetoma, chromoblastomycosis, and sporotrichosis, but often requires several months to years of therapy depending on the severity of infection. Itraconazole is often unavailable or unaffordable in many countries including in South-East Asia, particularly in rural areas where disease is more common.^6^^,^^53^^,^^55^^,^^56^ Additionally, concerns about the quality of generic itraconazole have been reported which could result in inadequate therapy.^57^^,^^58^ Alongside population surveys, regional pooled-procurement mechanisms in South-East Asia for high-quality itraconazole or other antifungals may improve access and treatment success while decreasing the cost by ministries of health to provide to the public. The Pan American Health Organization's Strategic Fund, which is a regional pooled-procurement mechanism, includes itraconazole and could serve as a model for other regions like South-East Asia.^59^ Improving access to adjuvant pharmacological and non-pharmacological therapy (e.g., cryotherapy, imiquimod) may be able to decrease duration of antifungals needed and pill burden by patients and health care systems.^60^^,^^61^

## Conclusion

Fungal NTDs are diseases of public health concern in South-East Asia but have been overlooked in the 2024–2030 South-East Asia Region's Regional Strategic Framework for NTDs. Clinicians, researchers, and public health professionals can collaborate on determining more accurate estimates of disease burden in communities and making these publicly available for policy makers at the local, national, regional, and global level. Public health active outreach including active case finding in communities can eliminate severe disease through the empowerment and training of community health workers in the context of integrated skin-NTDs programs.

## Contributors

DS conceptualized and wrote the original manuscript draft. TC and AC provided supervision. DS, HS, NP, MG, AMC, TC, PP, TB, AC reviewed and edited the manuscript.

## Disclaimer

The opinions expressed by the authors do not necessarily reflect the opinions of the Centers for Disease Control and Prevention (CDC) or the institutions with which the authors are affiliated.

## Declaration of interests

We declare no competing interests.
